# Explaining Osteomyelitis and Prosthetic Joint Infections (PJI) in terms of Biofilm – A Review

**DOI:** 10.5704/MOJ.2107.001

**Published:** 2021-07

**Authors:** S Singh, CL Tan, AR Ahmad

**Affiliations:** Department of Orthopaedics, International Medical University, Seremban, Malaysia

**Keywords:** biofilm, osteomyelitis, infected orthopaedic implants, prosthetic joint infections

## Abstract

Osteomyelitis is a chronic infection of bones. Eradication of bone infection is usually with antibiotics and debridement, but it is slow and the infection can recur even after many years. It is now established that osteomyelitis is due to biofilm and a better understanding of the process is required. We review the development of biofilm and apply it to osteomyelitis management. The planktonic microbes' response to adverse conditions is the formation of biofilm. Bacterial infections in planktonic forms cause infections that can be controlled with antibiotics and immunisation, however the same microbe when its phenotype becomes biofilm is more resilient. The understanding of how planktonic bacteria convert to biofilm is one of the aims set out for this article.

## Introduction

This review is to update the reader on the available data on biofilm. A pilot review done among academics and students in a medical college showed large gaps in knowledge on the topic. Osteomyelitis was described as an entity in 1852 by Edouard Chasseignac. John Hunter described the sequestrum in 1746. Antonie van Leeuwenhoek first detected biofilm from scrapings of his teeth and described them as animalcules.

Implant surgery is now an integral part of modern orthopaedics and the norm in orthopaedic surgery, from the management of fractures to the replacement of joints in degenerative joint disease and the substitution of bones in limb salvage surgery. The future also holds promise for several implantable (artificial intelligence) devices in orthopaedics, general surgery and other medical conditions. In the United States alone the number of joint replacements is estimated to exceed one million and the infection rate is said to be two percent, up to 20,000 infections per one million per year^1,2^. The use of internal fixation is rising as the number of fractures involving high-velocity injuries increases and these often require internal fixation with implants.

The indications for internal fixation of open fractures is increasing as grade 2 fractures are being fixed at initial surgical debridement and 3a fractures are being included for delayed internal fixation (Gastilo and Anderson classification). It includes the fact that internal fixation converts a closed fracture to an open grade 3a fracture with its inherent infection risks (Gastilo and Anderson classification)^3^. Presently local antibiotics are being used to prevent biofilm infection at a local concentration of 1000 times the minimum inhibitory concentration (MIC). This is the concentration required to eradicate biofilm, but at these concentrations, antibiotics have serious adverse reactions and cannot be used systemically.

Malaysia is reported to have the third highest fatality rates from road traffic accidents in Asia. A good percentage of fractures are open, and many fractures requiring implant surgery get infected with ensuing chronic osteomyelitis.

## Inclusion Criteria

Review articles, meta-analysis, and laboratory research articles on the developments in biofilm were searched from the years 2010 to 2020. Only articles in the English language were used. The search engines used were Pubmed and Google Scholar.

## Rationale for Review

Biofilms are collections of microorganisms that stick to non-biological surfaces, biological surfaces as well as each other. These groups of bacteria are often enveloped in an outer polymer layer produced by the microorganism or by the colonised host.

Many microorganisms to survive in diverse environments convert their phenotype to a sessile form^4^. An understanding of biofilm is essential for us to control this form of microorganism, as 90% of bacteria exist in this phenotype and if we want to control communicable diseases and infections we need to know biofilm morphology. Recent evidence reveals that biofilms are structurally complex dynamic systems that have both the characteristics of primitive multicellular organised organisms and complex ecosystems. Biofilm have been isolated from various conduits in hospital including drainage conduits of ICUs and respirators and their tubing^5^.

## Properties of Biofilm

Biofilm can vary, from a single cell layer to a thick collection of microbes which are surrounded by a thick polymeric milieu. The thick biofilms are arranged in distinct pillar or mushroom-shaped structures. An intricate network of channels provide access to nutrients from outside the biofilm to the deepest areas of the biofilm. Biofilm is typified by cells in a matrix attached to a surface or its interface, or to each other^6^. The matrix known as extracellular polymeric substance (EPS) is mainly composed of polysaccharides, eDNA, and also proteins secreted by cells within the biofilm, during its life cycle^7^. Biofilms organise themselves and can communicate, due to their close cell to cell proximity, and through the use of certain secreted proteins, nucleic acids, nano wires, and electrolytes through channels within the matrix.

Electron exchange by electrolytes through channels within the matrix can generate electricity which can and is commercially exploited^8^. They demonstrate the excretion of waste through channels and pores. Efflux pumps at the surface couple with antibiotics and allow for longer exposure to enzymes that degrade the antibiotics and reducing their efficacy e.g. β-lactamase for penicillins and catalase for peroxides^9^.

Biofilm cells change their nucleic composition, by transfer of nucleic material which can rapidly produce phenotypes to combat any adverse condition. This genetic material is transferred from cell to cell using F pilus, which are tubes that can contract and draw DNA material and transfer from one cell to another up to about 12 μm and can occur within seconds^5^.

Resilience: Most biofilms grow in adverse oligotrophic conditions with decreasing gradients of oxygen saturation, nutrients, and metabolites from superficial to deep, and this gradient makes the biofilm resilient against adverse conditions. This resilience is directly transferred by nucleic material from cell to cell by eDNA. This free-floating DNA which is derived from other cells that have ruptured can transfer laterally to other cells thus conferring resistance and immunity to other cells in the biofilm^5^.

Symbiotic existence: Metabolites of one species may be the nutrients of another species within the same biofilm and allows various species to live synergistically. The available nutrients in a matrix are in the form of polysaccharides metabolised to produce lactate by fermentation or, sulfides, carbon dioxide (CO2), hydrogen (H2) and methane (CH4). These metabolites in the extracellular polymeric substance (EPS) are freely exchangeable between cells^7^.

Microbes switch from planktonic to sessile states under the influence of c-d-GMP which is secreted due to stimulus from the environment leading to phenotypic, metabolic and physiologic changes. Adhesions occur and when quorum numbers (this is the minimum number of organisms required) are attained, EPS is also secreted under the influence of c-d-GMP and creates the sessile form of the biofilm accordingly^10^.

The slowdown of metabolic activities that can lead to slowing down of the movement of antibiotics and increasing the chance of it being degraded is due to longer exposure and increasing its resistance to the antibiotic. These events have led to the discovery of substances to penetrate the biofilm matrix and destroy it. These are collectively known as lantibiotics and are different from antibiotics as they act on biofilm, not bacteria.

## Biofilms and Cyclic Di-GMP(c-di-GMP) Signalling

The cyclic di-GMP (c-di-GMP) as a second messenger represents a signalling system that regulates many bacterial behaviours and is of key importance for driving the lifestyle switch between motile loner cells and biofilm formers. The bacterium, *Pseudomonas aeruginosa*, is frequently adopted as a model organism to study and illustrate bacterial biofilm formation.

Biofilm life cycle is the survival kit of biofilm. Through propagation, attachment and adhesions (under the influence of surface adhesins, autolysin A Protein-protein interactions, and conditioned surface interactions) it reaches the quorum number, that will give rise to secretion of EPS, and the formation of biofilm.

This is followed by the maturation phase and ends in the dispersion phase when the biofilm is seeded to remote places and activated by accessory gene regulator *(agr)*. The agr system in staphylococcus aureus, the most common commensal, is activated by auto inducing peptide(aip), leading to aggregation and dispersion once the quorum number is reached^11^. Quorum is a process which brings about the collection of bacteria which now behave collectively for the common good of the community.

C-di-GMP is an intracellular regulator of the phenotype from planktonic cell to sessile formation giving rise to a highly controlled community. To illustrate this point P. aeruginosa biofilms are estimated to contain on average 75–110pmol of-c-di-GMP per mg of total cell extract, whereas planktonic cells contain less than 30pmol mg1, demonstrating the concentration of c-di-GMP is directly linked to the activity of the bacterium^12^.

C-di-GMP controls properties of flagella rotation, exopolysaccharide production, surface adhesin expression, antimicrobial resistance and other stress responses to adverse conditions like secondary metabolite production, and biofilm dispersion^13^. This is a developmental process that includes an attachment to, and movement on the surface to the formation of micro-colonies, maturation, and ultimately dispersal^11,13^.

## Properties of Biofilm Attributable to the Matrix

Planktonic and biofilm are two forms of the same microorganism that transform from a motile to a sessile state and vice versa.

It is suggested 90% to 99% of bacteria live in biofilm form and the rest in planktonic form and 80% of chronic infections are due to biofilm. They have certain fundamental characteristics or traits which maybe common. For example, biofilm have channels of fluid running through it, resembling a circulatory system; it has the ability to respond protectively and its behaviour can be equated to an organism with a nervous system as it responds to internal and external threats. Pathogenic biofilm present a challenge for eradication by the host immune system and by chemotherapeutic agents^12,14^. A pathogenic biofilm is one that maybe an opportunistic pathogen which is more virulent as compared with commensal biofilms. Benign or commensal biofilms protect the human body from infection and disease, i.e. colonisation resistance.

Planktonic and biofilm have distinct features with the earlier causing acute infections, whereas biofilm correlate with chronic infections with resistance to both innate host immunity and `antimicrobial agents.

## Recalcitrance

There is a gradient of oxygen that develops between the outer layers and the interior. The centre has a lower concentration of oxygen due to the consumption of oxygen and the excretion of carbon dioxide and there is no recuperation of oxygen consumed. This makes the biofilm more recalcitrant and resistant to adverse environments.

Biofilm-embedded microorganisms benefit from certain advantages over their planktonic counterparts. These advantage can be listed as (a) the property of the extracellular matrix to seise and concentrate environmental nutrients, (b) the resistance to elimination by antimicrobial and antifouling agents, (c) shear stress, (d) host phagocytic clearance, (e) host oxygen radical and protease defences. This innate resistance to antimicrobial factors is due to the very low metabolic rate and radically down-regulated rates of cell division of the deeply entrenched microorganisms allowing the microbiols to denature these agents, (f) biofilms too may act as a diffusion barrier slowing down the infiltration of some antimicrobial agents. For example, reactive chlorine species (such as hypochlorite (Hibitane/chlorhexidine), chloramines, or chlorine dioxide) found in several antimicrobial/antifouling agents may be deactivated in the surface layers of the biofilm before they can disseminate into the deeper layers. In another study, alginate (a component of P. aeruginosa exopolysaccharide) was shown to be able to induce an alpha-helical conformation in antimicrobial peptides and likely entraps these peptides, preventing their diffusion into the biofilm, (g) the final benefit of the biofilm manner of growth is the potential for dispersion and propagation via detachment with subsequent seeding in a new area. Microcolonies may detach under the direction of the mechanical fluid shear force or through a genetically programmed response that mediates the detachment process^6^.

## Quorum Sensing and Adherence

Initially the bacteria’s attachment is mediated by passive non-specific forces namely hydrophobic, electrostatic, and Van der Waal forces, which work between close proximity of bacterial surfaces. Attachment is very rapid and can be achieved within minutes. Later they also create pili and fimbriae to gain attachment which grow in a matter of hours. The adherence is mediated through adhesins; polysaccharide intercellular adhesins (PIA) which consists of a glycosaminoglycan and are also considered virulence factors for staphylococcus aureus. These lead to the adhesion of cells to form a biofilm.

Biofilm is created when a quorum number is achieved using a quorum sensing mechanism that aggregate bacteria. Once the quorum number is achieved, the biofilm acquires all the qualities that make it distinct from planktonic bacteria^5^.

Despite these advances in understanding quorum sensing at the molecular level, Kavanaugh and Horswill emphasise that very little is known about how quorum sensing functions in the human environment. Host factors such as serum components, reactive oxygen species (ROS) and low pH are likely to play an important role^11^.

## Recalcitrance and Persisters, Glycocalyx-Enclosed Microcolonies Adhere to the Bone and Prosthetic Devices in Cases of Osteomyelitis

The refractory nature of biofilms such as seen in chronic infections and osteomyelitis, endows these bacteria to become persisters^15^. These persisters or recalcitrance, acquired by the bacteria make them resistant to host immunity and to antimicrobials and lead to chronicity of the infection.

Pathogenic biofilms possess greater numbers of upregulated genes, which in turn produce degrading enzymes, like, matrix metalloproteinases, enhance development of EPS, and enhance generation of quorum-sensing molecules, increase microbial proliferation, and microbial dissemination. These changes in biofilm lead to chronic inflammation that is unique to each microbe, hence each form of biofilm requires different management strategies for eradication^5^. So there is no one size fits all, and this is the reason a lot of chronic infections are said to be resistant to antibiotics.

Newer and more innovative techniques need to be developed to counter these problems. The only way to do that is to first find out why and how is the microbe resistant to antimicrobials or to the host immunity.

It is already been reported that concentrations of antimicrobial agents required for the eradication of in biofilm bacteria are more than 50 to 1000 times higher than those needed for the killing of the free-floating planktonic cells.

Biofilm is intimately linked with prosthetic joint infections. The materials commonly used in orthopaedic surgery consist of stainless steel, titanium polymethylmethacrylate cement, cobalt- chromium, and various other polymeric materials which have surfaces susceptible to attachment by biofilm. These implants develop a protein layer on their surfaces onto which the biofilm gets attached and goes on to form a glycocalyx.

Staphylococci comprise a diverse genus of Gram-positive, non-motile commensal organisms that inhabit the skin and mucous membranes of humans and other mammals. In general, Staphylococci are benign members of the natural flora, but many species can become opportunistic pathogens (with upgraded genes), mainly affecting individuals who have medical device implants or are otherwise immunocompromised. Staphylococcus generally are introduced in the implantation process directly through the wound or sometimes by intermittent bacteraemia (hematogenous spread) and then become pathogenic. The bacteria adhere to the implant and go on to form a biofilm15. During the colonisation phase of biofilm formation, bacteria alter their pattern of gene expression and should be regarded as interactive communities. They are not multicellular organism as they do not differentiate^16^.

Sub populations of bacteria evolve into phenotypically different resistant states expressing varied biofilm-specific antimicrobial resistance genes. Other bacteria within the biofilm may produce enzymes and exotoxins which result in local tissue invasion leading to the formation of fulminant infection. The reduced growth rate of bacteria due to accumulation of waste products, incomplete penetration of metabolic substrates together with mechanical and osmotic challenges in penetrating a biofilm makes the biofilm-based bacteria more resilient^17^.

Small colony variants are characterised by slow growth, decreased pigment formation, low coagulase activity, reduced hemolytic activity, and resistance to antibiotics^11^. Small colony variant bacteria can persist within host cells and it has been suggested that the intracellular location of this subpopulation might shield them from host defences and antibiotics. This is one explanation forwarded as to why chronic osteomyelitis can reactivate years after the initial infection^18^.

The final stage in the evolution of a biofilm involves the dispersion of planktonic bacteria. Through quorum sensing, gene expression(c-di-GMP) may alter the bacterial phenotype from colonising to invasive and as environmental conditions deteriorate within the biofilm, and bacteria disperse to find a surface with a more favourable environment.

## Method of Visualisation of Biofilm

The electron microscope, which allows high-resolution photomicroscopy at much higher magnifications than the light microscope brought in a new phase in the understanding of the structure and function of biofilm. Jones *et al.* used scanning and transmission electron microscopy to examine biofilms on trickling filters in a wastewater treatment plant and showed them to be composed of a variety of organisms, based on cell morphology^5,19^.

Besides electron microscopic techniques such as transmission electron microscopy, scanning electron microscopy and environmental scanning electron microscopy, modern fluorescence microscopic approaches based on fluorogenic dyes offer detailed insight into bacterial biofilms and an investigation of the genes involved in cell adhesion and biofilm formation.

Confocal microscopy is a specialised form of standard fluorescence microscopy (also called widefield fluorescence microscopy) that uses particular optical components to generate high-resolution images of material stained with fluorescent probes. Confocal microscopy differs from conventional widefield fluorescence microscopy in that the optical path of the laser light is designed to place in front of the image detector, an aperture at a point where the image is focused in conjunction with the focal plane of the image. This aperture removes all other light bringing the image into sharp focus without distracting light. These images are put together to form a full image and by controlling the depth of penetration of the laser, 3-dimensional images can be formed. This method of viewing bacteria has helped to view biofilm and understand their behaviour since 199920.

## Chronic Osteomyelitis

This is an infection of bone where the main causative microorganisms are sessile, rendering them less sensitive to systemic antibiotic agents and making routine culture techniques unreliable. The discovery that osteoclastic and osteoblastic cells play a central role in the immune response of bone has resulted in a better understanding of osteoimmunology. The knowledge of biofilm has provided new and effective means of understanding osteomyelitis and to fine-tune and enrol newer methods for the management of bone and prosthetic joint infections.

Osteomyelitis associated with vascular insufficiency occurs among individuals with diabetes mellitus and peripheral vascular disease. The infection at the surface is absorbed into the bone causing a sequestrum to separate (Wagner’s grade 3). New bone, or involucrum, then begin to form over the injured periosteum; and surround a sequestrum with ongoing drainage of pus (sinus) from the infected sequestrum^3^. As a consequence, the entire bone maybe invaded and remains chronically infected unless efforts are made to surgically and pharmacologically contain the infection. The outpouring of pus will find its way through a passage of least resistance to the skin surface and discharge as a sinus often known as a cloaca. Patients confined to a bed or wheel chair are subject to pressure-related skin ulceration and necrosis, most commonly in the sacral and buttock areas and these trophic ulcers then penetrate bone to cause osteomyelitis. These ulcers are frequently invaded by polymicrobial commensals which become pathogenic. These are forms of osteomyelitis induced by localised ischemia. These ulcers with osteomyelitis also occur in weight -bearing diabetic foot ulcers.

Microbial factors and biofilms are pathognomonic of chronic osteomyelitis. The S.aureus digested by osteoblasts can persist (persisters) and become more resistant to antimicrobials. It is also thought to block the inhibition of proteolysis in musculoskeletal structures^13,21^. The features of chronicity are dependent on the formation of sequestrum, involucrum, the age of the patient. Acute infections usually resolve with the use of antibiotics without requiring debridement.

## Classification of Osteomyelitis

Two systems are widely applied to osteomyelitis, the Cierny and Mader classification and the Waldvogel’s osteomyelitis classification system (Table I and II)^14^.

**Table I:** Cierny and Mader’s classification of osteomyelitis

Anatomical type	
*Type*	*Characteristics*
I	Medullary osteomyelitis
II	Superficial osteomyelitis
III	Localised osteomyelitis
IV	Diffuse osteomyelitis
**Physiological class**	
*Type*	*Characteristics*
A	Good immune system and delivery
B	Compromised locally (BL) or systematically (Bs)
C	Requires suppressive or no treatment; Minimal disability; Treatment worse than disease; Not a surgical candidate
**Factors affecting physiological**	**class**
*Systemic factors(s)*	*Local factors (l)*
Malnutrition	Chronic lymphedema
Renal or hepatic failure	Venous stasis
Diabetes mellitus	Major vessel compromise
Chronic hypoxia	Arteritis
Immune disease	Extensive scarring
Extremes of age	Radiation fibrosis
Immunosuppression	Small-vessel disease
Immune deficiency	Neuropathy
Tobacco abuse	
Alcohol abuse	
Malignancy	

**Table II:** Waldvogel classification of osteomyelitis

Anatomical type	
I	Medullary
II	Superficial
III	Localised
IV	Diffuse
**Physiological condition**	
A	Healthy
B	- Systematically compromised, Bs
	- Compromised locally, B1
	- Systematically and locally compromised, Bls
C	The majority of damage is due to treatment rather than disease
Factors influencing immunity, metabolism, and local blood supply	
**Systemic factors (Bs):** Malnutrition, chronic renal failure, liver failure, diabetes mellitus, chronic hypoxia, neonate/elderly, malignancy, immunosuppression or immune deficiency	

## Principles of Management

Both the systems (Fig. 2) use the anatomical and physiological methods to classify osteomyelitis. Chronic bone infections are biofilm-related and cannot be eliminated with IV antibiotics alone as the MIC provided is insufficient to eliminate the biofilm. In chronic osteomyelitis, the duration of antibiotics is governed by the organism, its resistance status, the healing status of the patient which is gauged by monitoring parameters like ESR, CRP, and radiological changes. The use of collagen impregnated with gentamicin up to 500-1000 times the MIC and the use of antibiotic-impregnated methyl methacrylate beads which slowly leach out the antibiotics are being used locally in the area of infection, after debridement. The use of local antibiotics is new but has proven to eliminate infections more effectively than the use of parenteral antibiotics by achieving higher levels of antibiotics locally. Implants coated with antibiotics and nanoparticles are being used now and will in the future be used more often. Covalent immobilisation of titanium with enoxacin by covalent bonding is another development to watch^22^. Debridement is the physical elimination of biofilm and is an essential part of managing osteomyelitis. Previously with the use of antibiotics alone, there was a failure of treatment in 30% of cases but with the advent of vascularised grafts it had dropped to 10-15%. Bone and tissue specimens must be sent for culture and sensitivity with positive cultures in 60% to 70% of samples as compared to 40%, from swab cultures as biofilm cultures cannot be obtained from swab cultures^23^. Rarely we find Pseudomonas especially in drug addicts sharing needles and using intravenous drugs. This technique has further improved our results in identifying the correct organisms.

## Modalities to Investigate Osteomyelitis

Technetium scans 99Tc for reactive bone and 67Ga, Gallium for inflamed and infected bone can be used in confirming the diagnosis. Today computerised tomography (CT) scans for pre-operative planning and magnetic resonance imaging (MRI) or Positron Scan for intra and extraosseous involvement are used in the diagnosis of osteomyelitis.

Nanoparticles are presently being used to breach the cell walls of the biofilm and the microbes4,24. Size is important here for the size of these nanoparticles helps in breaching the cell wall. Other properties of importance are the hydrophilic properties, penetration of the acidic matrix medium, and being able to bind with cationic ligands to penetrate cell walls as biofilms anions don’t bind as well as cations. All these have been observed with the use of confocal electroscopy^16^.

## Prosthetic Joint Infections

Prosthetic joint surgery is increasing at a tremendous pace and by the year 2040 is projected to increase by 401% for TKA and 284% for THR^25^. The readmission rate for joint replacement surgery ranges from 4% to 8% and often due to loosening of the prosthesis, periprosthetic fractures and infections. The recognition of infection is by wound dehiscence, discharge of purulent material, sinus opening, a warm swelling of the wound or scar area or if the surgeon suspects there is an infection.

Musculoskeletal Infection Society (MSIS) criteria for periprosthetic joint infection (PJI) in 2011 has resulted in improved diagnosis confidence and results. The recommend investigations and interpretation of research in prosthetic joint infections have resulted in recommendations in the use of antibiotics, debridement and the replacement of an infected prosthesis. There is also the consideration that as joint replacement surgery becomes popular so will the number of older patients with concomitant comorbidities. Biofilm tends to grow rapidly with compromised host immunity.

The isolation of the bacteria is more accurate when cultures are taken from biopsy material from synovial tissue and ultrasonication increases the yield. The prior history of the patient to antibiotic resistance to the delayed healing of wounds in the patient's history should be investigated.

The initial investigations will include radiographs, ESR CRP, leucocyte counts and CT scan. MRI bone scans and PET scans must all be done to plan the surgery.

Precision medicine initiative states "an emerging approach for disease treatment and prevention that takes into account individual variability in genes, environment and lifestyle for each person." This statement is to tailor the management to the needs of the patient and stop the “one size fits all” mentality. All these initiatives by the MSIS hope to improve the results of prosthetic joint infections. By bringing together recommendations, based on evidence obtained from research, with individual patient’s condition and profile we can individualise treatment with good predictability of the outcome.

The cornerstones of PJI is early identification of infection, antibiotics, debridement, removal of implant (the timing being dependent on the severity of infection) and reinsertion of an implant with or without bone grafts . These may require the cooperation of multiple physicians from plastic, vascular, infectious disease experts and imaging department.

As we can see these are chronic infections of biofilm origin and thus have high resistance to host immunity and various methods of management has been elucidated earlier.

## Summary

The study of osteomyelitis and implant infection is better understood now after the elucidation of biofilm as the cause of chronic bone infection. There is still some way to go to understand and eradicate implant infections. The use of multiple strategies starting with the recognition of the slimy appearance of biofilm to the use of mechanical removal of biofilm by debridement, the eradication of dead space, and lastly to the required concentrations of antibiotics that are needed to eradicate biofilm will help to reduce the incidence of osteomyelitis.

We now also have the use of lantibiotics, nanoparticles, inhibition of the exchange pump mechanisms and inhibition of neutralising enzymes that are found in the matrix. With a system of precise medicine in place we should strive to improve our study and understanding of both the patient and biofilm. It is a changing world with an increasing geriatric population.

The future will require a better understanding of biofilm formation and its life cycle of adhesion, adherence, maturation and dispersion to manage chronic infections.
